# TLR2/4 signaling pathway mediates sperm-induced inflammation in bovine endometrial epithelial cells *in vitro*

**DOI:** 10.1371/journal.pone.0214516

**Published:** 2019-04-17

**Authors:** Mohamed Aboul Ezz, Mohamed Ali Marey, Ahmed Essam Elweza, Tomoko Kawai, Maike Heppelmann, Christiane Pfarrer, Ahmed Zaky Balboula, Abdelmonem Montaser, Kazuhiko Imakawa, Samy Moawad Zaabel, Masayuki Shimada, Akio Miyamoto

**Affiliations:** 1 Graduate School of Animal and Food Hygiene, Obihiro University of Agriculture and Veterinary Medicine, Obihiro, Japan; 2 Department of Theriogenology, Faculty of Veterinary Medicine, Mansoura University, Mansoura, Egypt; 3 Department of Theriogenology, Faculty of Veterinary Medicine, Damanhur University, Behera, Egypt; 4 Department of Theriogenology, Faculty of Veterinary Medicine, University of Sadat City, Sadat City, Minoufiya, Egypt; 5 Graduate School of Biosphere Science, Hiroshima University, Higashi-Hiroshima, Japan; 6 University of Veterinary Medicine Hannover, Hannover, Germany; 7 Institute of Agricultural Sciences, Tokai University, Kumamoto, Japan; INSERM, FRANCE

## Abstract

We have recently shown that sperm attachment to bovine endometrial epithelial cells (BEECs) triggers uterine local innate immunity with induction of a pro-inflammatory response *in vitro*, however details of the mechanism remain unknown. Here, we investigated the involvement of Toll-like receptor 2/4 (TLR2/4) pathway in mediating sperm-BEECs inflammatory process. Immunohistochemistry of the uterine tissue revealed that TLR2 and TLR4 proteins were present in the luminal and glandular epithelia of bovine endometrium. Moreover, BEECs monolayers were treated with TLR2 agonist (Pam; 0, 10, 100, and 1000 ng/ml) or TLR4 agonist (LPS; 0, 0.1, 1, and 10 ng/ml) for 0, 1, 3, or 6 h, followed by evaluating mRNA expression of the pro-inflammatory genes (*TNFA*, *IL-1B*, *IL-8*, and *PGES*) in BEECs using a real-time PCR. Both Pam and LPS treatments showed a dose-dependent stimulation of mRNA expression of the pro-inflammatory genes. To elucidate the functional role of TLR2/4 in sperm-BEECs interaction, BEECs monolayers were incubated with either TLR2 antagonist or TLR4 antibody for 2 h prior to the co-culture with sperm for 3 h. Importantly, pre-incubation of BEECs with TLR2 antagonist or TLR4 antibody prevented the stimulatory effect of sperm on the transcription of pro-inflammatory genes in BEECs. Furthermore, sperm increased the phosphorylation levels of TLR2/4 downstream targets (p38MAPK and JNK) in BEECs within 1 h of the co-culture. Treatment of BEECs with TLR2 antagonist prior to sperm addition inhibited JNK phosphorylation, while TLR4 antibody inhibited the phosphorylation of both p38MAPK and JNK. In conclusion, the present *in vitro* findings strongly suggest that bovine endometrial epithelial cells respond to sperm *via* TLR2/4 signal transduction.

## Introduction

Although millions of sperm are deposited into the bovine uterus during artificial insemination (AI), most of these sperm are eliminated and only a few thousands reach to the oviduct where fertilization takes place [1]. The deposited sperm is considered antigenic to the uterine innate immune system and so induce an inflammatory response. This inflammatory response is characterized by a rapid and transient leukocytic infiltration, mostly polymorphonuclear cells (PMNs), into the uterine lumen which removes dead or in excess sperm cells [2].

The uterus and in particular the endometrium orchestrates many pivotal functions such as the conceptus implantation, placentation, and maintenance of pregnancy until normal parturition [3]. Concurrently with these reproductive functions, the uterus requires a defense system against invading pathogens as well as tolerating allogenic sperm and semi-allogenic embryo. Therefore, the uterine milieu should be equipped with a well-developed and strictly controlled immune system that can respond effectively to various antigens to which it is exposed [4].

To fulfill these requirements, uterine endometrial cells develop a mucosal innate immunity as well as their capacity to control the recruitment and activity of immune cells of both innate and adaptive immune functions [5]. The innate immune response, which has been considered to be the first line of defense against invading pathogens, depends on germ-line-encoded pattern recognition receptors (PRRs) to distinguish “infectious non-self” from “non-infectious self” [6]. PRRs are innate immune cell receptors involved in the initiation of inflammatory responses to either infections or sterile tissue injuries through recognition of highly conserved molecules called pathogen-associated molecular patterns (PAMPs) or damage-associated molecular patterns (DAMPs) respectively. In the last few decades, special attention has been paid to Toll-like receptors (TLRs) which are PRRs that can detect any changes in the micro-environment of the cells that possess these receptors [7].

TLRs are membrane-spanning proteins with extracellular domains of leucine-rich repeats. At least 11 mammalian TLRs have been identified with each receptor having its specific ligand [8]. Gene transcripts of TLRs 1 to 10 have been detected in the bovine endometrium where epithelial cells express TLRs 1 to 7 and 9, and stromal cells express TLRs 1 to 4, 6, 7, 9 and 10 [9]. The relevance of TLRs, especially TLR2 and TLR4, in the bovine endometrium has become apparent because of their ability to sense and react to lipopeptides of Gram-positive bacteria (by TLR2) and lipopolysaccharides (LPS) of Gram-negative bacteria (by TLR4) that may invade the uterus around the time of coitus or parturition [10]. Besides their involvement in the induction of inflammatory responses towards pathological agents as a part of the defense mechanism, emerging evidence suggests that TLR2/4 pathway participates in the inflammatory cascades responsible for important physiological processes such as ovulation [11] and fertilization [12].

Recently, we demonstrated that live, but not dead, sperm attach to BEECs *in vitro* which subsequently produce an acute inflammatory response [13]. Nevertheless, the mechanism whereby sperm induce such inflammatory response in BEECs was still unclear. Here, we investigate the possible involvement of TLR2/4 signaling pathway in mediating sperm-BEECs inflammatory process *in vitro*.

## Material and methods

### Ethics statement

All experiments described in this article were conducted in accordance with the Guiding Principles for the Care and Use of Research Animals Promulgated by Obihiro University of Agriculture and Veterinary Medicine, Japan. The protocol was approved by the Committee of Ethics of Animal Experiments of Obihiro University of Agriculture and Veterinary Medicine (Permit number 27–74).

### Uterine sampling

Uterine samples for immunohistochemistry were collected from six Holstein Friesian cows at the Cattle Clinic of University of Veterinary Medicine, Hannover, Germany). Simply, blood samples were collected from the jugular vein into serum tubes (Sarstedt, Nürnbrecht, Germany) for progesterone (P4) analysis. Afterwards, the cows were euthanized and the uterine horns including both ovaries were collected from each cow *via* a transverse incision in the ventral abdomen. The ovaries were examined for the presence and size of corpora lutea (CL) and follicles, while the serum P4 concentration was measured using a commercial radioimmunoassay kit (P4 Coat-a-Count, TKPG1, Siemens Medical Diagnostics, CA, USA) according to the manufacturer’s instructions. Cows with a CL and/or a serum P4 concentration > 1 ng/ml were assigned to the group “luteal phase”, while cows without CL and/or a serum P4 concentration < 1 ng/ml and a follicle diameter ≥ 14 mm were assigned to the group “follicular phase”. From both groups (3 cows per each group), inter-caruncular tissue samples (1.0 cm x 0.5 cm) including all layers were taken from the dorsal part of the uterine horns.

Uterine samples for the isolation and culture of BEECs were collected from the local slaughterhouse (Hokkaido Livestock, Doto Plant Tokachi Factory; Obihiro, Hokkaido, Japan). The bovine uterine horns (contra-lateral to mature follicle) were carefully incised and macroscopically examined to be free from any abnormal findings. The phase of the estrous cycle was determined based on the appearance, weight, and color of the corpus luteum and follicular diameter as previously reported [14]. Only healthy uterine horns (ipsi-lateral to mature follicle) of the pre-ovulatory phase (Days 19–22) were selected, dipped in physiological saline containing 1% penicillin-streptomycin (Gibco, Grand Island, NY, USA) and 1% amphotericin B (Gibco), and transported to the laboratory within 1–1.5 h on ice.

### Immunohistochemistry

The uterine tissue samples were fixed in formalin and embedded in paraffin, then immunohistochemistry was employed [15]. Briefly, the tissue was sectioned at 3 μm, mounted on silane-treated glass slides (Histobond Superior; Paul Marienfeld Laboratory Glassware, Laud-Königshofen, Germany) and dried at 37°C for 24 h. To block endogenous peroxidase activity, the sections were treated with 2% hydrogen peroxide in 80% alcohol solution for 30 min. To avoid non-specific binding sites, the sections were incubated with 20% normal goat serum at room temperature for 20 min, followed by incubation with primary antibodies (rabbit anti-TLR2 [1:100; orb11487, Biorbyt, Cambridge, UK] or rabbit anti-TLR4 [1:200; 251111, Abbiotec, San Diego, USA]) in a humid chamber overnight at 4°C. According to the datasheets and the immunohistochemistry protocols both antibodies were used at a concentration of 5 μg/ml. For detection of the immunoreaction, an EnVision anti-rabbit immunoglobulin conjugated to peroxidase-labeled dextran polymer system (DAKO, Glostrup, Denmark) was used in accordance with the manufacturer’s protocol. Finally, DAB (Sigma-Aldrich, Steinheim, Germany) was used as chromogen (5 min at room temperature), followed by counterstaining with hemalum, dehydration and mounting with DPX (Fluka, Buchs, Switzerland). Negative controls were done by substituting the primary antibodies with a rabbit IgG (Sigma-Aldrich) at the same concentrations.

### BEECs isolation and culture

The uterine horn ipsilateral to mature follicle was used for isolation and culture of epithelial cells following the protocol previously described [16, 17] with minor modifications. Briefly, epithelial cells were dissociated and suspended in Dulbecco's Modified Eagle Medium: Nutrient Mixture F12 (DMEM/F12) (Gibco) supplemented with 22 mM NaHCO_3_ (Sigma-Aldrich), 0.1% gentamicin (Sigma-Aldrich), 1% amphotericin B (Gibco), and 10% heat-inactivated fetal calf serum (FCS) (Bio Whittaker, Walkersville, MD, USA). After that, cells were seeded in 25 cm^2^ culture flasks (Nalge Nunc International, Roskilde, Denmark), and cultured at 38.5°C in a humidified atmosphere of 5% CO_2_ in air until reached 70–80% confluence. Then, cells were passaged, trypsinized, and re-seeded (at 1 x 10^5^ cells/ml) in 1.5 ml/well culture medium (DMEM/F12, 22 mM NaHCO_3_, 0.1% gentamicin, 1% amphotericin, and 5% FCS) in 12 well plates (Nalge Nunc International) until sub-confluence. These plated, BEECs were primed with 50 pg/ml estradiol-17β (E_2_) (Sigma-Aldrich) and 1 ng/ml P_4_ (Sigma-Aldrich) for 3–4 days until the end of each experiment. The concentration of each steroid hormone was based on levels measured in the bovine uterus during the pre-ovulatory period *in situ* [18, 19]. The purity of the cultured epithelial cells was evaluated through their characteristic epithelial morphology and confirmed with immunofluorescence staining using a monoclonal antibody against cytokeratin (anti-cytokeratin 8+18; ab53280, Abcam, Tokyo, Japan) [13].

### Sperm preparation

Frozen 0.5 ml semen straws were obtained from three highly fertile Holstein bulls belonging to Genetics Hokkaido Association, Hokkaido, Japan, where semen collection and processing were conducted under strictly controlled hygienic measures and the frozen-thawed semen was proven to be free from any bacterial contaminants. Nine straws (three straws from each bull) were thawed in a water bath at 38.5°C for 30 sec, pooled, and washed 3 times using a Tyrode’s albumin, lactate, and pyruvate medium (Sp-TALP) [20]. Progressive motility of the recovered sperm was assessed by visual examination using a light microscope equipped with a stage warmer; it was consistently around 50%.

### Co-culture of BEECs with sperm

Sub-confluent (around 80%) BEECs monolayers in 12-well plates were incubated for 2 h, as an equilibrium period to regain their activity, in 1 ml/well culture medium consisting of DMEM/F12, 22 mM NaHCO_3_, 0.1% gentamicin, 1% amphotericin, 0.1% FCS as well as E_2_ and P_4_ at the above mentioned concentrations. After 2 h of pre-incubation, BEECs monolayers were co-cultured with the 10^6^ sperm/ml for 1or 3 h. The dosing and timing of sperm-BEECs co-culture were selected on the basis of our previous results [13].

### TLR2/4 activation or blockage

To activate TLR2/4 pathway in BEECs, sub-confluent BEECs monolayers in 12-well plates were exposed to TLR2/4 agonist for 0, 1, 3, or 6 h (the same time window used for sperm-BEECs co-culture).

Pam3Cys-Ser-(Lys)4 (Pam; ab142085, Abcam) was used (at 10, 100, and 1000 ng/ml) as TLR2 agonist [21], while or LPS from *E*. *coli O55*:*B5* (Sigma-Aldrich) was used (at 0.1, 1, and 10 ng/ml) as TLR4 agonist for TLR4 activation [22].

For blockage of TLR2/4 pathway in BEECs, sub-confluent BEECs monolayers in 12-well plates were incubated with TLR2/4 blocker for 2 h. TLR2 antagonist (CU-CPT22; Merck, Darmstadt, Germany) was used (at 0.05 μM = 18.12 ng/ml, and 0.1 μM = 36.24 ng/ml, and 0.5 μM = 181.20 ng/ml) as TLR2 blocker [23], while TLR4 antibody (MAb2-hTLR4; InvivoGen, San Diego, CA, USA) was used (at 10, 100, and 1000 ng/ml) as TLR4 blocker [24].

### RNA extraction, cDNA synthesis, and quantitative real-time PCR

RNA was extracted from the cells using Trizol reagent (Invitrogen, Carlsbad, CA, USA) as previously reported [25]. Extracted RNA was quantified using a NanoDrop Spectrophotometer 2000c (Thermo Scientific, Waltham, MA, USA), and the purity of each sample was assessed by the ratio A_260_/A_280_. Pure preparations of RNA (A_260_/A_280_ ratio between 1.8 and 2.0) were stored in RNA storage solution (Ambion, Austin, TX, USA) at -80°C until cDNA synthesis.

The cDNA synthesis was done following the previously described protocol [26] with minor modifications. First, a DNase treatment step was performed using RQ1 RNase-Free DNase kit (Promega, Madison, WI, USA) to remove residual genomic DNA and other contaminants. Through which, 1 μg of extracted RNA was incubated for 30 min at 37°C in a thermal cycler (Eppendorf, Hamburg, Germany) with a first mixture consisting of 1 μl of RQ1 RNase-free DNase 10X Reaction Buffer, 2 μl of RQ1 RNase-free DNase (1 unit/ μl), and Nuclease-free water (Invitrogen, Carlsbad, CA, USA) to a final volume of 10 μl followed by addition of 1 μl of the RQ1 DNase Stop solution for 10 min at 65°C to terminate the reaction. After that, the first-strand cDNA was synthetized according to the commercial protocol described in the SuperScript II Reverse Transcriptase kit (Invitrogen). Simply, the DNase-treated RNA was incubated at 65°C for 5 min with a second mixture consisting of 1.5 μl of 3 μg/μl random primer, 1.5 μl of 10 mM PCR Nucleotide Mix (dNTP) (Roche Diagnostics, Indianapolis, IN, USA) and Nuclease-free water to a final volume of 18 μl. In this sequence, a third mixture consisting of 6 μl of 5X First-Strand Buffer, 3 μl of 0.1M dithiothreitol and 1.5 μl of 40 units/μl Ribonuclease Inhibitor Recombinant (Toyobo, Osaka, Japan), was added per each tube and then incubated at 42°C for 2 min followed by the addition of 0.2 μl of 200 units/μl SuperScript II Reverse Transcriptase and the thermal cycler was programmed at 25°C for 10 min, 42°C for 50 min and then 70°C for 15 min. The synthesized cDNA was stored at -30°C.

The mRNA expression levels of our selected genes: Tumor necrosis factor (TNF) alpha *(TNFA)*, Interleukin (IL)-1 beta *(IL-1B)*, *IL-8*, and Prostaglandin E synthase *(PGES)* were determined by a quantitative real-time polymerase chain reaction (PCR) using an iQ5 real-time PCR detection system (Bio-Rad Laboratories, Tokyo, Japan). Simply, a total 10 μl reaction mix consisting of 2 μl/sample synthesized cDNA, 5 μl of QuantiTect SYBR Green PCR Master Mix (QIAGEN, Hilden, Germany), 0.2 μl of the targeted primer pairs (listed in Table 1), and 2.8 μl nuclease-free water (Invitrogen) was prepared. Afterwards, the amplification program was run with an initial denaturation step at 95°C for 15 min, followed by 40 cycles of denaturation at 95°C for 15 sec, annealing at 51–55°C (according to each primer) for 30 sec, extension at 72°C for 20 sec. A negative control, reactions containing nuclease-free water or non-reverse transcribed RNA were included in each run. The primers pairs were designed by Primer Express Software v3.0.1 (Thermo Scientific). The efficiency of the qPCR amplifications for B-actin, TNFA, IL-1B, IL-8 and, PGES mRNAs were 101.67, 93.88, 92.69, 96.08, and 107.71%, respectively. The calculated cycle thresholds (Ct) were exported to Microsoft^®^ Office Excel, normalized using *ACTB* (*β-actin*), and the delta-delta comparative threshold method was used to quantify the fold change between the samples [27]. The housekeeping gene, *β-actin*, was used as an internal standard for normalization of Ct values because its mRNA expression was stable in all experiments; no significant variations were observed in its mRNA expression across the different treatments [13, 26].

**Table 1:** List of primers used for real-time PCR.

Gene	Forward primer	Reverse primer	Accession No.	Product size (bp)
***B-actin***	5'-TCACCAACTGGGACGACATG	5'-CGTTGTAGAAGGTGTGGTGCC	NM_173979.3	51
***TNFA***	5'-CAAAAGCATGATCCGGGATG	5'-TTCTCGGAGAGCACCTCCTC	NM_173966.3	51
***IL-1B***	5'-AATCGAAGAAAGGCCCGTCT	5'-ATATCCTGGCCACCTCGAAA	NM_174093.1	51
***IL-8***	5'-CCAATGGAAACGAGGTCTGC	5'-CCTTCTGCACCCACTTTTCCT	NM_173925.2	51
***PGES***	5'-AAAATGTACGTGGTGGCCGT	5'-CTTCTTCCGCAGCCTCACTT	NM_174443.2	51

### Western blotting

Protein samples from BEECs were prepared by homogenization in cell lysis buffer (cOmplete Lysis-M EDTA-free, Roche, Switzerland) with PhosSTOP (Roche) and then diluted in the same volume of Sample Buffer Solution with 2-ME (2x) (Nacalai Chemical, Osaka, Japan) for SDS-PAGE. After ultrasonic treatment, protein samples were incubated at 100°C for 5 min. Extracts were separated by SDS polyacrylamide gel (10%) electrophoresis and transferred to polyvinylidene fluoride membranes (GE Healthcare Life Sciences, PA, USA). Non-specific sites were blocked using a solution of 5% (w/v) BSA (Sigma-Aldrich) in Tris-buffered saline and Tween 20 (TBS-T, 10 mM Tris, pH 7.5,

150 mM NaCl, and 0.05% Tween 20). Membranes were then incubated with a primary antibody (listed in Table 2) overnight at 4°C. After washing in TBS-T, the membranes were incubated with a secondary antibody [1:5000 dilution of goat anti-rabbit IgG horseradish-peroxidase-labeled antibody (Cell Signaling Technology, Beverly, MA, USA) or 1:5000 dilution of goat anti-mouse IgG horseradish-peroxidase-labeled antibody (Cell Signaling Technology) diluted in 5% (w/v) non-fat dry milk in TBS-T] for 1.5 h at room temperature. After washing in TBS-T, chemiluminescence detection was performed using an ECL Prime Western Blotting Detection Reagent (GE Bioscience) according to the manufacturer’s specifications, followed by appropriate exposure of the blots to Fuji X-ray film (Fujifilm, Tokyo, Japan). The intensity of the bands was analyzed using a Gel-Pro Analyzer (Media Cybernetics, Rockville, MD, USA). All primary antibodies were used at 1:1000 dilutions. Meanwhile, secondary antibodies for β-actin and total ERK 1/2 were used at 1:5000 dilutions and other secondary antibodies were used at 1:3000 dilutions.

**Table 2:** List of antibodies used for Western blotting.

Target	Antibody	Catalog No.	Species
**Phospho NFκB**	Anti-phospho-NFκB p65 (Ser536) (93H1) antibody	Cell signaling (3033S)	Rabbit
**NFκB**	Anti- NF-κB p65 (D14E12) antibody	Cell signaling (8242S)	Rabbit
**Phospho ERK1/2**	Anti- phospho-p44/42 MAPK (Erk1/2) (Thr202/Tyr204) (20G11) antibody	Cell signaling (4376S)	Rabbit
**ERK1/2**	Anti- p44/42 MAPK (Erk1/2) (L34F12) antibody	Cell signaling (4696S)	Mouse
**Phospho p38 MAPK**	Anti- phospho-p38 MAP kinase antibody	Cell signaling (9211S)	Rabbit
**p38 MAPK**	Anti-p38 MAP kinase (C-20) antibody	Santa Cruz Biotechnology (sc-535)	Goat
**phospho JNK**	Anti-phospho-SAPK/JNK (Thr183/Tyr185) antibody	Cell signaling (9251)	Rabbit
**JNK1**	Anti-JNK1 (FL) antibody	Santa Cruz Biotechnology (sc-571)	Rabbit
**Phospho IRF3**	Anti-phospho-IRF3 (S396)(4D4G) antibody	Cell signaling (4947S)	Rabbit
**β-actin**	Anti-β-actin monoclonal antibody	Sigma-Aldrich (A5316)	Mouse

### Statistical analysis

Each experiment was repeated 3–4 times using epithelial cells from 3–4 different uteri. In each uterus, 3 replicates were performed (3 wells per treatment per experiment) and data are presented as mean ± standard error of the mean (SEM). The mean of the 3 replicates of each uterus was calculated and these values were used in the statistical analysis and shown in figures. Statistical analyses were performed using IBM SPSS Statistics 24 (IBM, Armonk, NY, USA). Student’s t-test was applied to compare the data between two groups, while one-way ANOVA followed by LSD post hoc multiple comparison test was used for more than two groups. The results were considered statistically significant at *P*< 0.05.

## Results

### Bovine endometrium expressed TLR2 and TLR4 proteins

**Fig 1:**
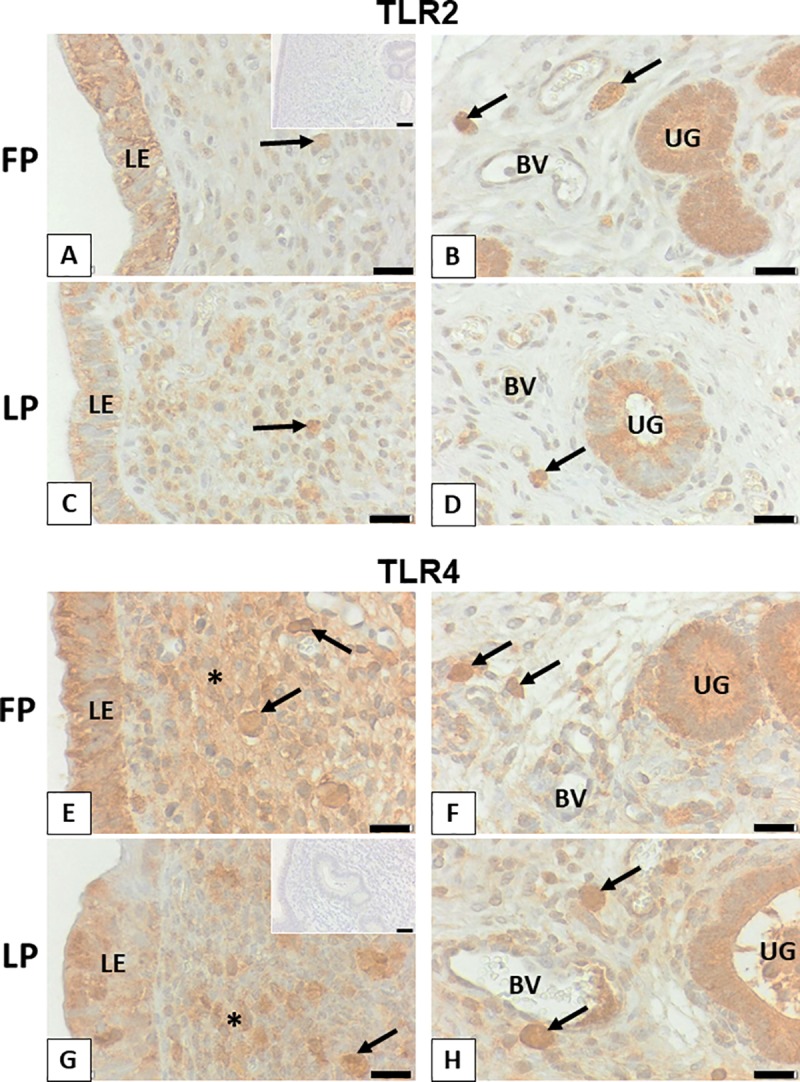
Bovine endometrium expressed TLR2 and TLR4 proteins. Representative sections for luminal epithelium and stratum compactum (A, C, E, G), as well as stratum reticulare with endometrial glands (B, D, F, H) and blood vessels (BV) are shown. TLR2 is localized in luminal (LE) and glandular epithelium (UG) as well as a few immune cells (arrows) during follicular phase (FP) and luteal phase (LP). TLR4 is found in the same cells and additionally in stromal cells of the stratum compactum (asterisks) in some areas. Scale bars, 20 μm from A to H. Insets to A and G show representative controls for TLR2 and TLR4, respectively with scale bars: 40 μm.

TLR2 and TLR4 immunoreactions were observed in the luminal and glandular epithelia of bovine endometrium during both follicular and luteal phases. Besides, a few immune cells were stained by both antibodies. TLR4 immunostaining was additionally localized in groups of stromal cells of the stratum compactum **(Fig 1).**

### TLR2/4 activation induced transcription of the pro-inflammatory genes in BEECs

**Fig 2:**
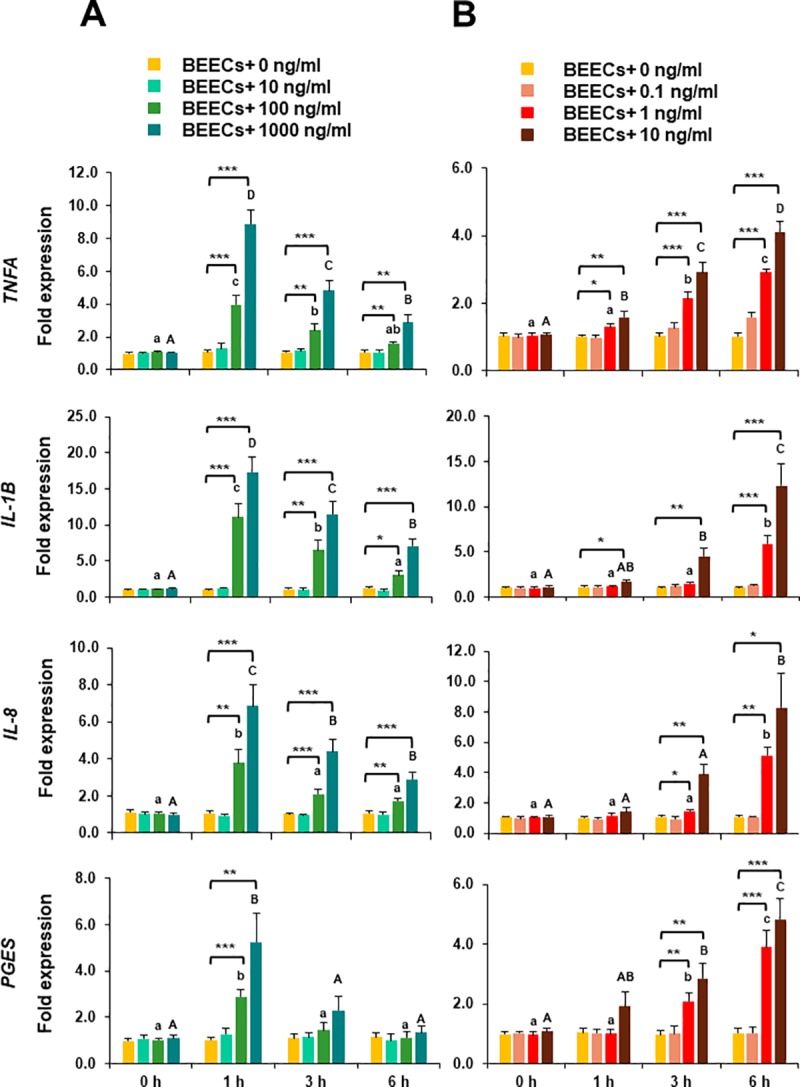
TLR2/4 activation induced transcription of the pro-inflammatory genes in BEECs. Sub-confluent bovine endometrial epithelial cells (BEECs) monolayers were exposed to A) TLR2 agonist (Pam; 0, 10, 100, and 1000 ng/ml) or B) TLR4 agonist (LPS; 0, 0.1, 1, and 10 ng/ml) for 0, 1, 3, or 6 h. At each time point, mRNA expressions of *TNFA*, *IL-1B*, *IL-8*, and *PGES* in BEECs were quantified by a real-time PCR. Data are presented as mean ± SEM of 4 independent experiments using epithelial cells from 4 different uteri (3 wells per treatment per experiment). Asterisks denote a significant variance [* (*P* < 0.05), ** (*P* < 0.01), *** (*P* < 0.001)] between the different doses of Pam/LPS when compared to the control group (0 ng/ml) at each time point. Different small letters denote a significant variance (*P* < 0.05) between the different time points of 100 ng/ml Pam (A) or 1 ng/ml LPS (B). Different capital letters denote a significant difference (*P* < 0.05) between the different time points of 1000 ng/ml Pam (A) or 10 ng/ml LPS (B).

TLR2/4 activation by Pam/LPS dose-dependently induced mRNA expressions of the pro-inflammatory cytokines (*TNFA* and *IL-1B*), chemokines (*IL-8*) and prostaglandins E synthesis (*PGES*) in BEECs. Specifically, the inflammatory response towards Pam peaked at 1 h, subsided towards 3 h and was still subsiding towards 6 h **(Fig 2A)**, while LPS inflammatory response was a time-dependently progressing **(Fig 2B)**.

### TLR2/4 blocker inhibited the inflammatory response of BEECs towards TLR2/4 agonist

**Fig 3:**
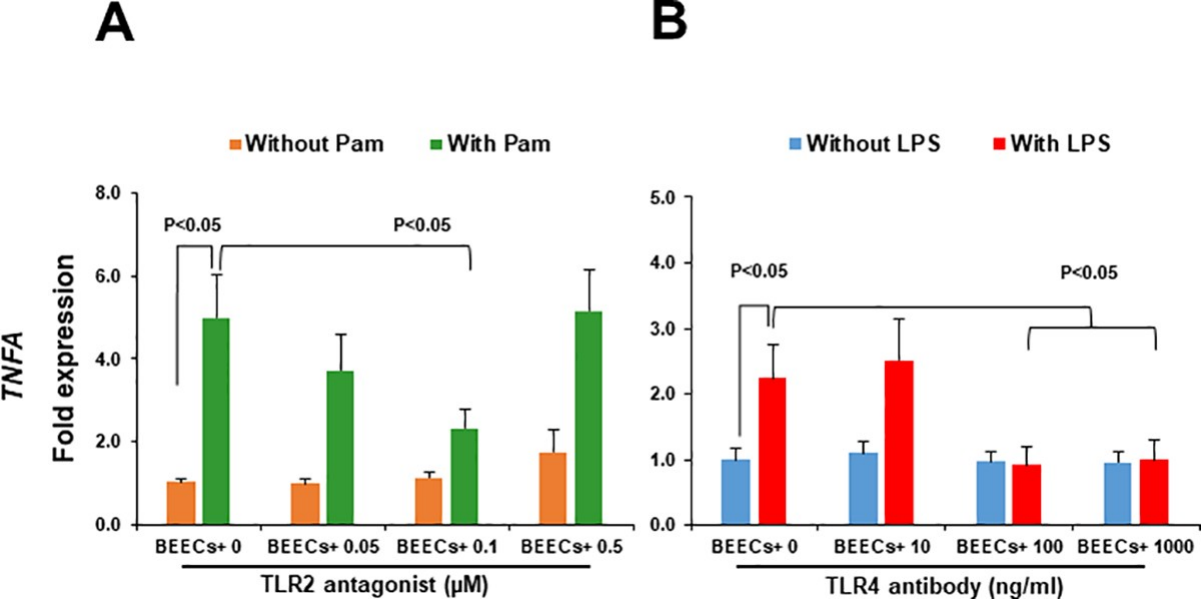
TLR2/4 blocker inhibited the inflammatory response of BEECs towards TLR2/4 agonist. Sub-confluent BEECs monolayers were 2 h incubated with A) TLR2 antagonist (0 μM, 0.05 μM = 18.12 ng/ml, and 0.1 μM = 36.24 ng/ml, and 0.5 μM = 181.20 ng/ml) prior to stimulation with Pam (100 ng/ml) for 1 h or B) TLR4 antibody (0, 10, 100, and 1000 ng/ml) prior to stimulation with LPS (1 ng/ml) for 3 h. *TNFA* mRNA expression was then quantified in BEECs. Data are presented as mean ± SEM of 3 independent experiments using epithelial cells from 3 different uteri (3 wells per treatment per experiment).

In order to detect the possible role of TLR2/4 in sperm-BEECs interaction, it was necessary to investigate whether TLR2/4 blocker can neutralize sperm-BEECs inflammatory response. Initially, an optimization experiment was conducted to test the efficacy of these blockers in our *in vitro* model. BEECs monolayers were pre-incubated (2 h) with different concentrations of TLR2 antagonist or TLR4 antibody then stimulated with Pam or LPS, respectively. We found that 0.1 μM of TLR2 antagonist or 100 ng/ml of TLR4 antibody was the minimal concentration that could inhibit the mRNA expression of *TNFA* in BECs in response to Pam or LPS, respectively **(Fig 3A and 3B)**. Accordingly, the above mentioned concentrations of TLR2 antagonist and TLR4 antibody were then used for TLR2 and TLR4 blockage in BEECs respectively prior to the co-culture with sperm.

### TLR2/4 blocker prevented the stimulatory effect of sperm on the transcription of pro-inflammatory genes in BEECs

**Fig 4:**
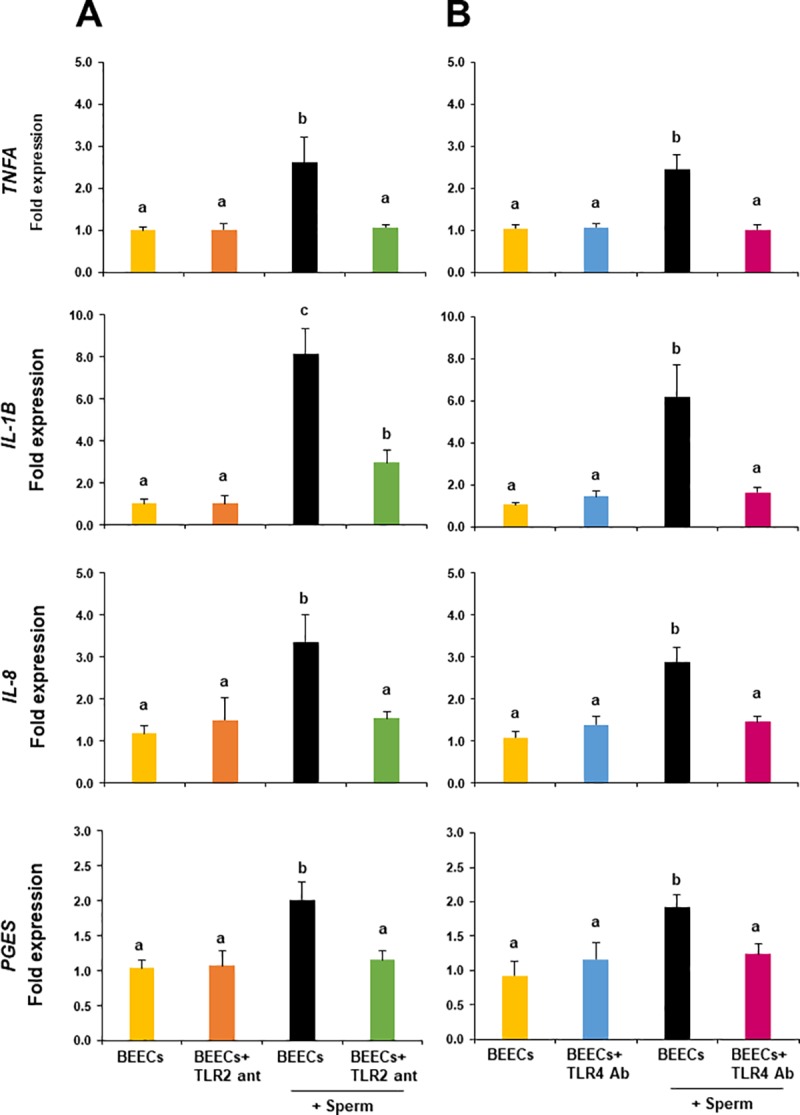
TLR2/4 blocker prevented the stimulatory effect of sperm on the transcription of pro-inflammatory genes in BEECs. Sub-confluent BEECs monolayers were incubated for 2 h with A) 0.1 μM TLR2 antagonist or B) 100 ng/ml TLR4 antibody, then stimulated with 10^6^/ml sperm for 3 h. mRNA expressions of *TNFA*, *IL-1B*, *IL-8*, and *PGES* were then quantified in BEECs. Data are presented as mean ± SEM of 4 independent experiments using epithelial cells from 4 different uteri (3 wells per treatment per experiment). Different letters denote a significant variance (*P*< 0.05) between the different groups.

To elucidate the functional role of TLR2/4 in sperm-BEECs interaction, BEECs monolayers were incubated with either TLR2 antagonist or TLR4 antibody for 2 h prior to the co-culture with sperm for 3 h. Our real-time PCR data showed that the pre-incubation of BEECs with 0.1 μM of TLR2 antagonist significantly suppressed the influence of sperm on the abundances of *TNFA*, *IL-1B*, *IL-8*, and *PGES* transcripts **(Fig 4A)**. Likewise, the stimulatory effect of sperm on the transcription levels of these genes was abolished by the supplementation of 100 ng/ml of TLR4 antibody to the sperm-BEECs co-culture media **(Fig 4B).**

**Fig 5:**
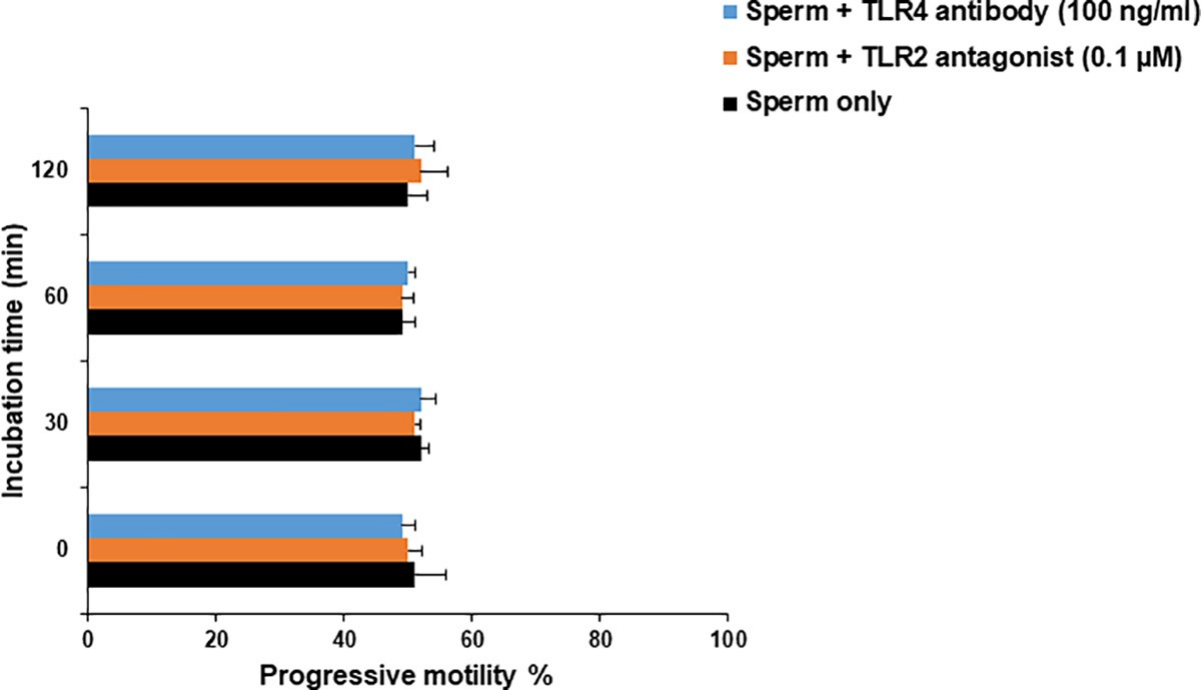
Direct effect of TLR2/4 blocker on sperm motility. Sperm cells at 10^6^/ml Sp-TALP were exposed to either 0.1 μM TLR2 antagonist or 100 ng/ml TLR4 antibody for 0, 30, 60, and 120 min. At each time point, progressive motility of recovered sperm from both groups was assessed and compared with those of the control group (without either TLR2 antagonist or TLR4 antibody). Data are presented as mean ± SEM of 3 independent experiments using epithelial cells from 3 different uteri (3 wells per treatment per experiment).

To exclude the possibility that TLR2/4 blocker could adversely affect sperm motility, sperm cells at 10^6^/ml Sp-TALP were exposed to either 0.1 μM TLR2 antagonist or 100 ng/ml TLR4 antibody for 2 h and progressive motility of the recovered sperm was assessed at 0, 30, 60, and 120 min. Importantly, TLR2/4 blocker did not affect progressive motility of the recovered sperm over the exposure time **(Fig 5)**.

### TLR2/4 blocker inhibited TLR signal transduction pathways in BEECs in response to sperm

**Fig 6:**
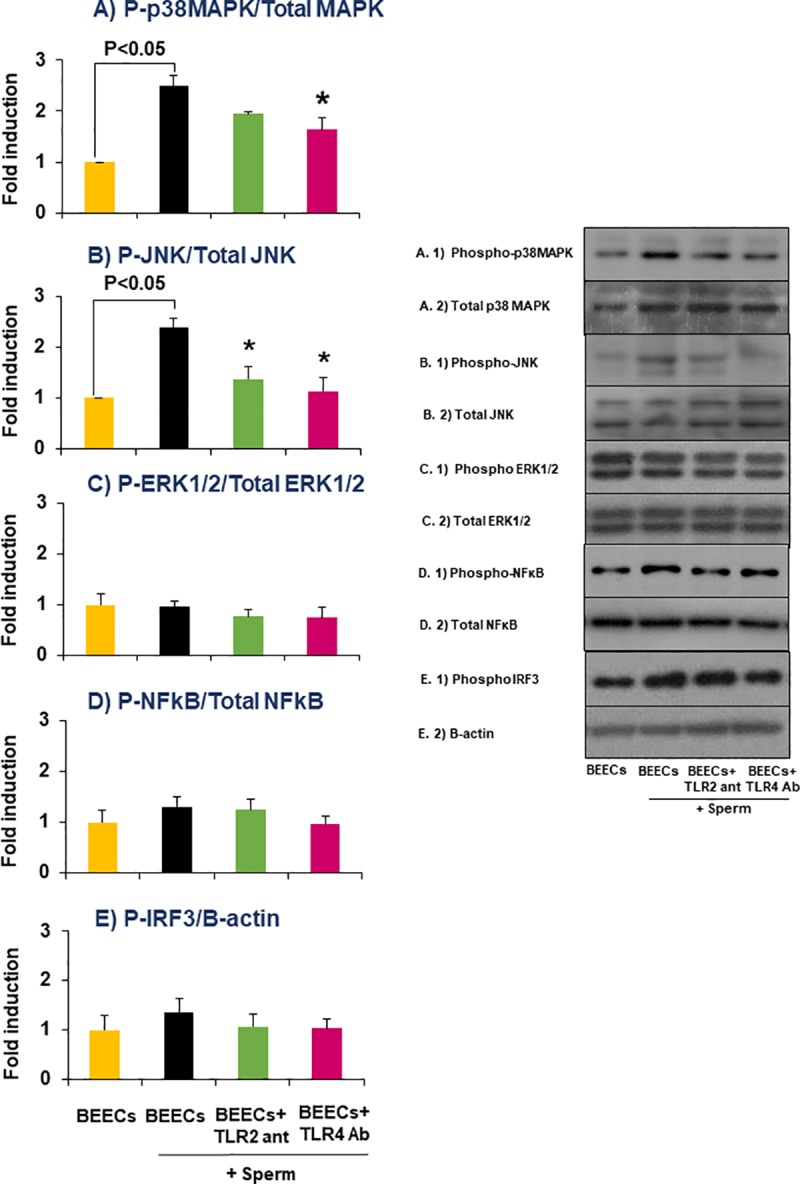
TLR2/4 blocker inhibited TLR signal transduction pathways in BEECs in response to sperm. Sub-confluent BEECs monolayers were incubated for 2 h with 0.1 μM TLR2 antagonist or 100 ng/ml TLR4 antibody, then stimulated with 10^6^/ml sperm for 1 h. Western blotting was then carried out to estimate the phosphorylation levels of A) p38MAPK, B) JNK, C) ERK1/2, D) NFkB, and E) IRF3 in the different groups. Data are presented as mean ± SEM of 3 independent experiments using epithelial cells from 3 different uteri (3 wells per treatment per experiment). Asterisk (*) denotes a significant variance (P< 0.05) between TLR2/4 blocking groups when compared with sperm group.

The activation of MAPKs (p38MAPK, JNK, and ERK1/2), NFkB, and IRF3 signaling was examined to seek further evidence for the involvement of TLR2/4 pathway in sperm-BEECs inflammatory process. Western blot analyses revealed a significant increase in the phosphorylation levels of both p38MAPK and JNK in BEECs within 1 h of the co-culture with sperm **Fig 6A and 6B)**, whereas the phosphorylation levels of ERK 1/2, NFkB or IRF3 did not vary significantly **Fig 6C, 6D and 6E)**. Additionally, the pre-treatment of BEECs with TLR2 antagonist significantly inhibited the sperm-induced phosphorylation of JNK, while TLR4 antibody significantly inhibited the phosphorylation of both p38MAPK and JNK in BEECs exposed to sperm.

## Discussion

The present study using a simplified co-culture model of endometrial epithelial cells and frozen-thawed sperm provides a novel finding that TLRs, specifically TLR2/4, activation pathway is involved in regulation of sperm-BEECs inflammatory process. The addition of TLR2/4 blocker to the co-culture media abolished the impact of sperm on the transcript abundance of pro-inflammatory genes through inhibiting the transduction of TLR signaling pathways in BEECs.

In a recent study, we documented that active sperm stimulate an inflammatory cascade in BEECs *in vitro* [13], but the mechanism of uterine epithelial cells to sense and react to sperm was still unknown. Inflammation is a cellular response stimulated by either infectious or non-infectious agents. TLRs represent a key molecular link between infection or tissue injury and the subsequent inflammatory response [28]. Of TLRs, we focused on TLR2 and TLR4 due to their ability to mediate inflammation not only at the interaction of bacteria with endometrial cells during infection [10], but also at the interaction of sperm with cumulus-oocyte complexes during fertilization [12]. In our study, TLR2 and TLR4 proteins are indeed expressed in epithelia of bovine endometrium with no clear difference in their expressions between follicular and luteal phases of the estrous cycle. These findings on the protein level support the earlier findings that detected gene transcripts of TLRs 1 to 7 and 9 in the bovine endometrial epithelial cells [9].

As a prerequisite for their functionality activated by bindings with a ligand, TLR2 and TLR4 in BEECs were ligated with their specific agonists (Pam for TLR2, and LPS for TLR4). Both Pam and LPS promoted inflammatory responses as expected in BEECs, but with different time-dependent manners. The different kinetics of inflammatory responses caused by Pam and LPS could be attributed to the mode of actions, levels of permeability, concentrations, and solvents used [29, 30]. These findings indicate that TLR2/4 pathway was functional in our *in vitro* culture model even though we used lower concentrations and shorter time windows than previously reported by others [31, 32].

The facts that TLR2 and TLR4 proteins localize in the epithelial cells of bovine endometrium as well as the capability of specific TLR2/4 agonist to activate a pro-inflammatory response in BEECs prompted us to explore the possible role(s) of TLR2/4 in sperm-BEECs interaction. In our previous study, sperm induced a rapid and transient pro-inflammatory reaction in BEECs. Definitely, sperm stimulated transcript abundances of the pro-inflammatory genes (*TNFA*, *IL-1B*, *IL-8*, and *PGES*) in BEECs with a maximum stimulation at 3 h. Moreover, the concentrations of pro-inflammatory cytokines (TNFA and IL-1B) in sperm-BEECs co-culture were under the limits of detection by ELISA [13]. It was suggested that release of such cytokines to extracellular matrix requires formation of an intracellular inflammasome as a consequence of an ongoing potential danger to cell viability [32]. Thus, the lack of protein accumulation probably reflects the weak and transient effect of sperm as a stimulus for inflammation in BEECs. Subsequently, the mRNA expression of pro-inflammatory genes was used as an indicator to evaluate the impact of TLR2/4 blockage on the extent of inflammatory response of BEECs towards sperm. Importantly, we found that the pre-incubation of BEECs with either TLR2 antagonist or TLR4 antibody reduced the magnitude of sperm-induced inflammation. These observations strengthen our notion that TLR2/4 activation pathway is involved in sperm-BEECs inflammatory process.

Two major signaling pathways can be activated *via* TLRs, MyD88-dependent pathway (which is recruited by TIRAP) and MyD88-independent pathway (which is recruited by TRAM) [33, 34]. Definitely, TLR2 engagement can only activate the MyD88-dependent pathway, while TLR4 engagement can activate both pathways [35]. The MyD88-dependent pathway involves the activation of IRAK4-TRAF6 complex which in turn activates either MAPKs (p38MAPK, JNK, and ERK1/2) or IKKs, and ends with the transcription of the pro-inflammatory genes *via* AP-1 or NFkB, respectively [36]. On the other hand, the MyD88-independent pathway results in the synthesis of interferon through the phosphorylation of IRF3 [37]. Accordingly, we examined the phosphorylation levels of p38MAPK, JNK, ERK1/2, NFkB, and IRF3 as downstream targets of the two major signaling pathways of TLR2 and TLR4 in BEECs. Our protein analyses showed that sperm enhanced both phospho-p38MAPK and phospho-JNK without any significant effect on the phosphorylation levels of ERK1/2, NFKB or IRF3 in BEECs. These outcomes raise the possibility that sperm activate TLR2/4 signaling pathway, mainly MyD88-dependent pathway, to induce a pro-inflammatory cascade in BEECs possibly through AP-1 activation. In the same direction, it was found that AP-1 (but not NFkB) activation through MAPKs (JNK) signaling was sufficient to drive a uterine inflammatory pathway that caused preterm labor in mice [38]. To ensure the former possibility, TLR2/4 blocker was incorporated into sperm-BEECs co-culture. The phosphorylation level of p38MAPK was suppressed by the addition of TLR4 antibody, while the phosphorylation level of JNK was significantly suppressed by the addition of either TLR2 antagonist or TLR4 antibody. Similarly, it was reported that stimulation of bovine endometrial cells by either bacterial lipopeptides or LPS induced a pro-inflammatory response *via* the activation of MAPKs pathway. In addition, MAPKs inhibitors suppressed the transcription of the pro-inflammatory genes in these cells [31, 32]. Therefore, these observations provide further support to the functional role of TLR2/4 in mediating sperm-BEECs inflammatory process.

Whereas the current study supplies several lines of evidence for the role of TLR2/4 signaling pathway in the response of endometrial epithelial cell to sperm, we cannot ignore the probability that endogenous ligands are involved. A wide variety of TLR2/4 endogenous ligands have been recently reported including heat shock protein-70 (HSP70) [39], high mobility group box-1 protein (HMGB1) [40], as well as extracellular matrix molecules like biglycan [41] and hyaluronan (HA) fragments [42]. Clearly, further investigations are essential to identify which molecule(s) link sperm to TLR2/4 signaling pathway for induction of such particular inflammatory response in BEECs.

**Fig 7:**
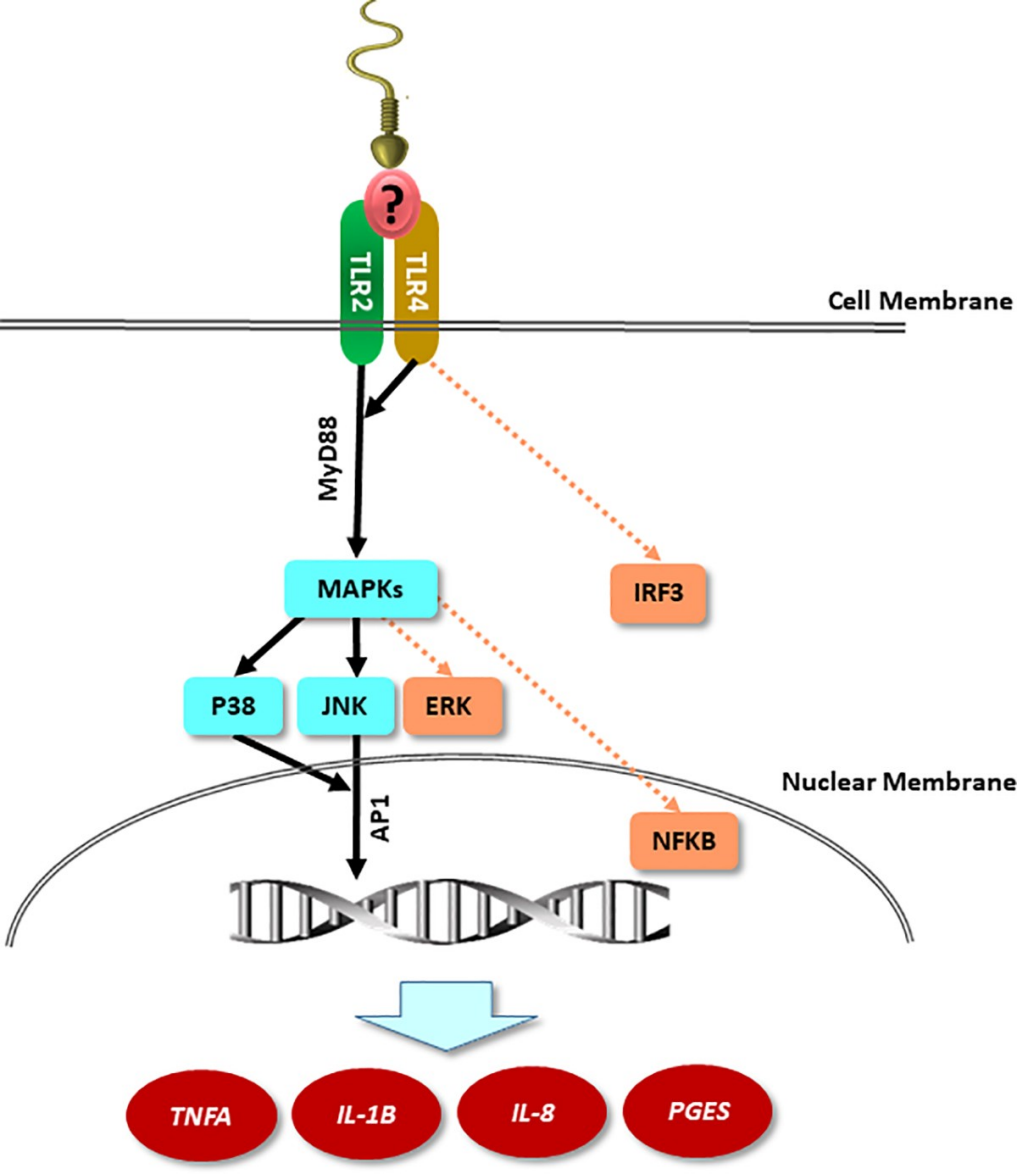
A diagrammatic illustration of the mechanism by which sperm activate TLR2/4 signaling pathway in BEECs *in vitro*. The proposed working model showing that stimulation of BEECs with sperm activates MAPKs components (p38MAPK and JNK; solid lines) without any effect on other downstream targets of TLR2/4 signaling pathways (ERK1/2, NFKB or IRF3; dotted line) down to the nuclear translocation of AP-1 protein which in turn regulates the transcription of pro-inflammatory genes in BEECs. “?” refers to unknown endogenous ligand(s) which may link sperm to TLR2/4 signaling pathway in BEECs.

Collectively, our data suggest that sperm use TLR2/4 signaling in a MyD88-dependent pathway to activate downstream components (p38MAPK and JNK) which should in turn stimulate nuclear translocation of the inflammatory transcription factor (AP-1) with subsequent transcription of pro-inflammatory cytokines (*TNFA* and *IL-1B*), chemokines (*IL-8*) as well as prostaglandin E synthesis (*PGES*) in BEECs *in vitro* **(Fig 7)**. In conclusion, TLR2/4 signal transduction in endometrial epithelial cells is involved in their response to sperm.
